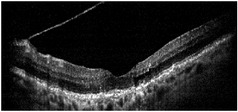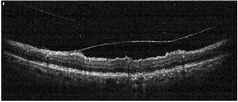# A Case of Secondary Epiretinal Membrane Spontaneous Release

**DOI:** 10.1155/2016/4925763

**Published:** 2016-10-31

**Authors:** Andrey N. Andreev, Alexey V. Bushuev, Sergey N. Svetozarskiy

**Affiliations:** ^1^Department of Ophthalmology, Volga District Medical Centre under Federal Medical and Biological Agency, Nizhnevolzhskaya Embankment 2, Nizhny Novgorod 603001, Russia; ^2^Department of Eye Diseases, Nizhny Novgorod State Medical Academy, Minin and Pozharsky Square, 10/1, Nizhny Novgorod 603005, Russia

## Abstract

*Purpose.* To report a rare case of secondary epiretinal membrane (ERM) spontaneous separation with subsequent visual restoration.* Case Summary.* We are reporting a case with the history of branch retinal vein occlusion, peripheral retinal neovascularization, and retinal photocoagulation. Our examination revealed secondary ERM associated with relatively high visual acuity (0.6), and a watchful waiting strategy was chosen. During the follow-up, slight visual deterioration, progressive deformation of the retinal profile, and an increase in diffuse retinal edema were observed. No surgical or laser treatment was performed. On the next visit, the spontaneous ERM separation with residual parapapillary fixation, the increase in visual acuity (0.9), and the decrease in retinal thickness were revealed.* Conclusion.* Such cases present additional evidence to a deferral surgical strategy for the management of patients with ERM and relatively high visual acuity.

## 1. Introduction

Epiretinal membrane (ERM) is a common disease of the vitreoretinal interface, presented by a sheet of avascular fibrocellular translucent tissue growing on the internal limiting membrane [1, 2]. ERM is classified as idiopathic when it is not associated with intraocular inflammation, retinal vascular diseases, trauma, retinal detachment, and retinal surgery and as secondary when it develops in connection with other ocular diseases [2, 3].

The main treatment strategy for ERM is surgical removal of the membrane during pars plana vitrectomy with internal limiting membrane peeling. Increase in visual acuity after the intervention occurs in 70% of idiopathic ERM cases, visual acuity decreases in 15%, and in 15% visual functions remain unchanged [4]. The greatest change in visual acuity following ERM surgery is revealed in patients with poorer preoperative visual functions [4]. Postsurgical ERM recurrence is observed in 1.3–16.5% [4–6], cataract occurs in 28% after ERM surgery, glaucoma is seen in 2.1%, retinal detachment develops in 0.8%, and phthisis bulbi is found in 0.4% [4]. Taking this into account, management of ERM patients with relatively high visual acuity (0.5 and above) currently remains controversial.

In this article we describe a rare case of natural history of the secondary ERM with the relatively high initial visual acuity.

## 2. Case Presentation

A 65-year-old woman came to the Volga District Medical Centre in March 2013 with symptoms of blurred vision in her left eye. She had a history of the branch retinal vein occlusion in the left eye in January 2012 and 2 sessions of retinal laser photocoagulation in the upper quadrants (February and April 2012). On examination, best corrected visual acuity (BCVA) was 0.7 in the left eye. The lens was clear and fundus showed pigment atrophy and retinal wrinkling in the macular area, occlusion of the upper-temporal branch of the central retinal vein, traces of laser photocoagulation in the upper quadrants, and peripheral retinal neovascularization with focal vitreous hemorrhages. Optical coherence tomography (OCT) (Optovue, RTVue-100) revealed incomplete posterior vitreous detachment, ERM on the macular surface, and slight diffuse retinal edema. The diagnosis was ERM, proliferative retinopathy with partial vitreous hemorrhage, condition after retinal photocoagulation, and mild myopia in the left eye. Watchful waiting strategy was chosen with three-month check-ups recommended.

During the follow-up from August 2013 to April 2014 there were not any new complaints; thus no laser or surgical treatment was carried out. There was moderate reduction in visual acuity (0.6), progressive deformation of the retinal profile due to the ERM tangential traction, increase in diffuse retinal edema, and gradual ERM separation (Table 1). The fundus showed gradual regress of peripheral retinal neovascularization and vitreous hemorrhage resorption.

In December 2015, the patient experienced vision improvement in the left eye. There was significant improvement of BCVA (0.9), appearance of translucent film upon the macula, and complete regression of peripheral retinal neovascularization in the left eye. According to OCT, ERM was separated from the macula surface with residual parapapillary fixation. On the next follow-up visit in February 2016, the visual functions were stable, the retinal profile improved, and the posterior hyaloid was still attached to the ERM surface (Table 1).

## 3. Discussion

ERM spontaneous separation is rare (3–6%) [7–9], and it is essential to observe this process in the dynamics. Spontaneous ERM release was described in association with toxoplasma uveitis, eye trauma, and laser surgery (pan-retinal photocoagulation and YAG-laser capsulotomy) [7, 10]. The case of the secondary ERM separation associated with branch retinal vein occlusion is presented in the literature for the first time.

Visual improvement after surgical treatment of secondary ERM associated with branch retinal vein occlusion was observed only in 48.5% of patients, and decrease was observed in 9.1% [11]. At the same time, visual functions in the majority of ERM patients with relatively high visual acuity (0.5 and above) remain stable for two years of observation in the absence of treatment, and visual decline occurs in less than 10% of the cases [9]. These data support the watchful waiting strategy chosen for the patient.

There are a few ERM separation mechanisms described [12]: rupture of weakest line provoked by lifting heavy objects (Valsalva), complete posterior vitreous detachment, and ERM remodeling due to myofibroblasts contraction. In our report, residual posterior hyaloid attachment to ERM, as well as continuous ERM structure without any defects, excludes the first two mechanisms of ERM separation. Myofibroblasts contraction is most likely to be the driving force of the process we have been observing for three years. The tangential traction led to the ERM transition from complete attachment to incomplete, foveal distraction and, as a result, caused the ERM separation. It is worth noting that the retina in fovea was spontaneously restored after several years of tractional deformation, and visual acuity improved significantly. This indicates a high degree of photoreceptors resistance to long-term distraction by ERM.

Management of ERM with relatively high visual acuity is still a matter of debate. The high efficacy of operation supports the early surgical strategy. At the same time, a high percent of surgically intact patients with stable visual functions, the risk of surgical failure, and ERM recurrence call for the watchful waiting strategy. Cases of spontaneous ERM separation with increasing visual acuity are rare, but along with the other evidence it calls for the regular OCT-based observation for the patients with ERM and relatively high visual acuity.

## Figures and Tables

**Table 1:** Best corrected visual acuity (BCVA), central retinal thickness, and OCT images of the patient's left eye during the three-year follow-up. ERM separation revealed in December 2015 was accompanied by visual acuity improvement and decrease in retinal thickness.

Date	BCVA	Central retinal thickness, *µ*m	OCT images
March 2013	0.7	273	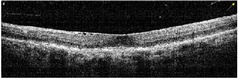

August 2013	0.6	326	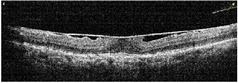

October 2013	0.6	339	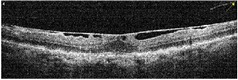

December 2013	0.6	372	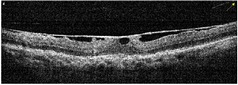

April 2014	0.6	391	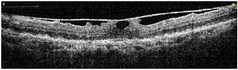

December 2015	0.9	236	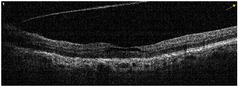

February 2016	0.9	123